# Offenders With Personality Disorder Who Fail to Progress: A Case-Control Study Using Partial Least Squares Structural Equation Modeling Path Analysis

**DOI:** 10.2196/27907

**Published:** 2021-10-29

**Authors:** Georgina Mathlin, Mark Freestone, Celia Taylor, Jake Shaw

**Affiliations:** 1 Centre for Psychiatry and Mental Health Wolfson Institute of Population Health Queen Mary University of London London United Kingdom; 2 Alan Turing Institute London United Kingdom; 3 Millfields Unit John Howard Centre East London Foundation Trust London United Kingdom; 4 Oxleas NHS Foundation Trust London United Kingdom

**Keywords:** offender, personality disorder, progression, prison, PLS-SEM, partial least squares structural equation modeling, mental health, OPD, offender personality disorder, proactive diagnosis, psychopathy, psychosis, mental health services, health services

## Abstract

**Background:**

Offenders with personality disorder can be challenging to engage and retain in treatment. The UK Offender Personality Disorder (OPD) pathway aims to proactively and responsively identify and engage offenders with personality disorder. However, a subpopulation of offenders on the pathway have been found to not be accepted into any OPD service and therefore fail to progress.

**Objective:**

This study aims to identify and describe offenders on the OPD pathway who fail to progress and to understand the causal drivers by which individuals fail to progress in the pathway.

**Methods:**

A sample of 50 offenders on the OPD pathway who had been refused from at least two OPD services (nonprogression group) were compared to 100 offenders accepted into OPD services (control group). Partial least squares structural equation modeling was used to model the causal factors involved in not being accepted into OPD services.

**Results:**

The path coefficients in the structural model showed that the most influential factor in nonprogression was attitude toward treatment (*β*=.41; *P*<.001; *f*^2^=0.25) alongside those with psychopathology (*β*=.41; *P*<.001; *f*^2^=0.25), specifically, psychopathy, psychosis, and co-occurring personality disorder.

**Conclusions:**

The findings of the study provide a basis of how to work with this population in the future to increase the likelihood of acceptance into OPD services.

## Introduction

### Personality Disorder

Personality disorder is an enduring mental disorder in which the individual’s pattern of inner experience and behavior deviates markedly from the expectations of their culture. It is associated with significant psychological distress, comorbid mental illness, difficulties with interpersonal relationships, premature mortality [1], and in some cases increased risk to others [2]. The Diagnostic and Statistical Manual of Mental Disorders classification system groups the 10 subtypes of personality disorder into three clusters based on some shared characteristics. Cluster A contains odd and eccentric personalities; cluster B dramatic, impulsive, and emotional personalities; and cluster C fearful and anxious personalities. Psychopathy is a particularly extreme form of personality disorder, characterized by antisocial behavior combined with a callous lack of empathy and the absence of guilt; psychopathic individuals show little concern for the suffering of others. The overall community prevalence of personality disorder in the United Kingdom is around 4.5% [2], while psychopathy is a relatively rare condition affecting around 0.6% of the household population [3].

The subtypes of personality disorder most commonly associated with offending are antisocial (in which criminal behavior and aggressiveness form part of the definition), borderline, narcissistic, and paranoid. Individuals in the community with cluster B personality disorders are more likely to have had a criminal conviction and to have served a prison sentence [2]. People with any personality disorder are also twice as likely to report being a victim of violence [2]. A systematic review of 62 surveys found a prevalence of personality disorder among prisoners of 65% [4] while that of psychopathy is nearly 8% of male UK prisoners [5].

It has long since been recognized that offenders with personality disorder can be challenging to engage with treatment [6]. The importance of doing so is underscored by the fact that these offenders have poor criminal justice outcomes [7], including increased risk of violence [8] and increased odds of repeat offending [9]. Subsequently, the implementation of interventions and treatment for offenders with personality disorder receives a lot of attention [10,11].

### Engagement With Treatment

Factors associated with offenders with personality disorder not engaging with treatment include personal characteristics such as young age [12], low education level [13], low occupation level and unemployment [13,14], juvenile convictions [15], and childhood emotional neglect [16]. Psychological need factors have also been identified and include having several personality disorder diagnoses [17], low levels of persistence [18], high levels of avoidance [19], and poor ego structure [18]. Lastly, there are some environmental factors, for example, less previous service use [12] and poor therapeutic alliance [20].

For those offenders who do begin treatment, the likelihood of dropping out is high. A systematic review identified the attrition rate for offenders with personality disorder to be 37% [18], with other estimates as high as 73% [21]. Reoffending outcomes are worse for offenders who do not complete treatment, compared to those who do not engage with any treatment, even when the initial risk of reoffending is similar [22]. Young offenders with lower education and occupation levels, poor social problem solving, low levels of persistence, and greater avoidance coping styles have been found to be more likely to drop out of treatment [18], which are similar characteristics related to nonengagement.

### The Offender Personality Disorder Pathway

Given these complexities, since the early 2000s, UK Government policy has focused on developing a more informed approach to the assessment, risk management, and treatment of this complex offender group. The Offender Personality Disorder (OPD) pathway was developed in 2011 following consultation on the earlier Dangerous and Severe Personality Disorder Programme [23]. The *pathway* is jointly managed through National Health Service (NHS) England and the Ministry of Justice, and aims to adopt a proactive and responsive approach to identifying and engaging offenders with personality disorder. As part of this approach, probation staff and NHS clinicians work together to identify offenders with a likely diagnosis of personality disorder early in their sentence using a screening algorithm [24]. The screening tool helps identify people with a likely diagnosis of personality disorder or those with personality disorder traits and considers risk factors such as the type of sentence, sexual or violent offending, and risk of harm alongside indicators of personality disorder including childhood difficulties, mental health difficulties, self-harm or suicide attempts, and challenging behavior. A case formulation is then developed and used to inform sentence planning. Within secure environments, services available to those on the OPD pathway include specialist prison-based therapeutic services and therapeutic communities (TCs), and psychologically informed planned environments (PIPEs) [25]. Those whose needs cannot be met within the criminal justice system (CJS) can be transferred to a specialist secure psychiatric hospital for treatment.

The OPD approach, although in its early years, has seen broadly encouraging results. A considerable proportion of cases screened in as eligible have either been referred to a specialist service or have made a progressive move. A progressive move would include acceptance into an OPD service, a step down in security category, or release into the community. Over 36,000 offenders have been identified as suitable, and as of 2017, a total of 3400 had engaged with pathway interventions [26].

However, several of the authors are clinicians working within the London Pathways Partnership (LPP), a consortium of NHS trusts delivering services within the OPD pathway, and are aware of several individuals that no OPD service, in prison or the NHS, is prepared to accept. Anecdotally, this seems to be for several reasons, for example, the nature or degree of risk posed; disagreement about diagnosis; the offender’s unwillingness to engage in a therapeutic intervention; or, linked to this, a perception of being *untreatable* [27] or *difficult* [28], both psychosocial labels with a complex causality. The nonacceptance of offenders into OPD services on the OPD pathway is likely related to issues of engaging high-risk offenders [5] and overcoming obstacles to treatment readiness [29].

Given that one of the key stated purposes of the OPD pathway is to “manage breakdown and failure...to support future progression” [30], it is important to understand why some offenders are not progressing to learn how pathway plans can adapt. The OPD pathway is informed from the “What Works?” literature [31], the risk needs responsivity (RNR) principles [32] and the Good Lives Model [29]. However, the RNR model has been criticized for not providing clear guidance for therapists for engaging offenders lacking in treatment readiness [33]. The responsivity principle of the RNR model may not currently be effectively implemented in the OPD pathway and contributing to the problem of offenders being referred but not accepted to numerous OPD services.

Furthermore, there are costs associated with the OPD pathway. For example, an OPD prison bed was costed at £50,000 (US $68,812) [34] at the program’s launch, compared to £37,000 (US $50,920) for a standard prison bed at the same time point [35]. Offenders who are screened into the OPD pathway need to be treated cost-effectively, and nonprogression is likely to be costly. Aside from the acute costs of the OPD pathway, engaging and treating offenders with personality disorder is intended to reduce recidivism [36], and subsequently overall criminal justice spending.

It is clear, that despite its successes with many, the pathway approach is not meeting the needs of a group of individuals. For clinicians to be more responsive to this subpopulation of offenders on the OPD pathway, the characteristics of this group and the reasons why they are not being accepted into treatment need to be better understood. Much about the characteristics of this specific group is unknown, making it difficult to rectify this situation. For the OPD pathway to be meeting the aim of working with the most complex and stuck cases in the CJS, a refined understanding of the potential causal drivers of a failure to progress through services is sorely needed.

### Aims

The aim of this study is to identify and review the characteristics of individuals who have been screened into the OPD pathway but failed to progress and for whom no clear pathway can be established. Four objectives were identified to achieve the aim:

1. Identify a sample of offenders that have not progressed on the OPD pathway from routine data sets
2. Describe the failure to progress (nonprogression) sample cases’ demographic, offending, and clinical characteristics alongside referral information to identify common features of this group
3. Use a complex regression model (partial least squares structural equation modeling [PLS-SEM]) to understand the causal drivers by which individual offenders and ex-offenders identified for entry into the OPD pathway fail to progress within the pathway
4. Suggest possible approaches to meeting the criminogenic and psychological needs of the nonprogression sample

## Methods

### Sample and Procedure

All participants in the study were under the supervision of the London area of the National Probation Service (NPS). The nonprogression sample was purposive and consisted of 50 male offenders, identified through professionals such as Offender Managers, LPP psychologists, and LPP forensic psychiatrists. Emails explaining the rationale and aims of the study requested names of offenders who had been declined from at least two OPD services. Cases discussed at the NPS London Division IPP Complex Case Panel were also included in the nonprogression group when they met the inclusion criteria. A case-control design, where cases (nonprogression sample) were compared to a random sample of controls (progression sample), was used. The broader caseload comparison group consisted of male offenders identified from the LPP case database through random selection (every 10th case). Based on suggested sample sizes for PLS-SEM models with at least 10 cases per path in the *busiest* exogenous variable [37,38], we oversampled the comparison group at a ratio of 2:1 (100 cases) to obtain a total sample size of 150, allowing for up to 15 paths per node.

The demographics of the nonprogression and comparison samples are discussed in detail in the Results section, as this addresses the second aim of the research to understand common characteristics of the nonprogression sample. The random selection of the control group should ensure this is representative of the broader LPP caseload. The nonprogression sample should be representative of offenders not being accepted into OPD services, as these cases were identified through a variety of professionals working with these offenders and a case panel designed to discuss offenders who were not progressing through the OPD pathway.

Once cases were identified, the electronic records of the offender were accessed. A specifically designed research schedule containing information on demographics, offending, institutional behavior, psychopathology, risk measures, previous treatments, current attitudes toward treatment, and referrals to OPD services was applied by trained research assistants to systematically collect the relevant data from probation record systems (nDelius and the Offender Assessment System [OASys]) [39]. OASys is designed to assess likelihood of reoffending, classify offending-related needs, identify risk of harm to the individual and others, link assessment to the supervision plan, measure change during the supervision period, and indicate needs for further specialist assessment. nDelius is a browser-based NPS case management system containing offender-related information.

Gathering data from secondary data sources could have led to errors in recording; however, each research schedule was completed and checked over to ensure errors were identified. The measures identified are all routinely recorded information and are used throughout the CJS to assess offender characteristics and risk.

The same research schedule was used for the nonprogression and control group. The data sets generated and analyzed during this study are not publicly available due to confidentiality agreements with the NPS.

### Ethics

Ethical approval for this research was granted by the NPS London Division.

### Measures

#### Demographics

Demographic data included date of birth, ethnicity, and current marital status, and was recorded from OASys.

#### Offending

To measure offending, the following variables were recorded: most serious index offence (and, if applicable, secondary and tertiary index offences), the victim of the index offence, previous offending, current sentence type, and security category and recall history. All variables were categorical and obtained from OASys.

#### Institutional Behavior

Institutional behavior was measured by recording the number of adjudications the offender had during their current or most recent prison sentence, if they had ever previously escaped, absconded, been in long-term or repeated segregation, or displayed institutional violence or misbehavior (eg, having contraband, drug use, or not following the prison regime). All variables were measured as “yes” or “no” and collected from NDelius and OASys.

#### Psychopathology

Psychopathology was measured by identifying any previous diagnosis or significant traits of personality disorder, psychopathy (a score of over 25 on the Psychopathy Checklist-Revised [40]), psychosis, learning difficulty, autism spectrum disorder, organic brain disorder, depression, substance misuse, posttraumatic stress disorder, and self-harm. This information was found in OASys and was recorded as “yes” or “no.”

#### Risk

Risk measures included OASys severe personality disorder screen score, the Offender Group Reconviction Scale (OGRS), and OASys Violence Predictor (OVP) 2-year score were recorded from the most recent OASys assessment, the Risk Matrix 2000 (RM2000) [41], and Historical Clinical Risk (HCR-20) [42]. The OASys severe personality screen score comprises 10 items, with a score of 7 or above triggering a further assessment of personality disorder. The OGRS is a static, actuarial risk assessment providing an estimate of the probability that offenders will be reconvicted within 2 years of release [43]. The OVP is an actuarial violence predictor combining static and dynamic risk factors [39]. The RM2000 [41] is a risk assessment for sexual offenders created to classify sexual recidivism and risk of reconviction for sexual or nonsexual assaults and was recorded if available. Out of the sample that had a sexual conviction (n=21), all had data for this variable. The HCR-20 is a structured professional judgement measure for violence risk; 83 people did not have an HCR-20 assessment on record (n=24, 48% missing in the nonprogression group and n=61, 40% in the progression sample).

#### Previous Treatment

To measure previous treatment, any historical engagement with treatment including offender behavior programs (OBPs), prison TCs, high secure OPD prison service, PIPEs, high secure or medium secure health, and community-based treatments were recorded. For the nonprogression group, it was recorded whether the participant has previously refused, completed, dropped out of treatment, or a combination of the aforementioned (eg, completing one OBP but refusing another). This information was obtained through the nDelius contact log and OASys section 11.

#### Attitudes Toward Treatment

Finally, the nonprogression group sample had their current attitude toward engaging with treatment recorded. This was identified from the OASys section, which discusses treatment motivation, and was categorized into refusing treatment, refusing treatment in a chosen service, refusing treatment in an available service, or unable to engage (with reasons).

### Analysis Plan

The first objective of the study was to describe the common features of the nonprogression group. To achieve this, summary tables displaying descriptive statistics of the sample were produced, and tests of differences (chi-square or *t* tests) between the nonprogression group and the control group were run.

To understand the causal drivers by which individual offenders identified for entry into the OPD pathway fail to progress, PLS-SEM analysis was used to model the factors involved in not being accepted into OPD services. PLS-SEM is a form of structural equation modeling (SEM) that estimates path coefficients or relationships between several latent variables using a probabilistic algorithm know as maximum likelihood estimation, as opposed to the covariance-based approach adopted in standard SEM. PLS-SEM was chosen because the method can cope with formative constructs, which enhances the understanding of the linear relationships in failing to progress on the OPD pathway and can accept noncontinuous variables. It was predicted that pathways to nonprogression would be complex, with many variables impacting the relationship, so PLS-SEM was chosen over a simpler regression model.

**Figure 1:**
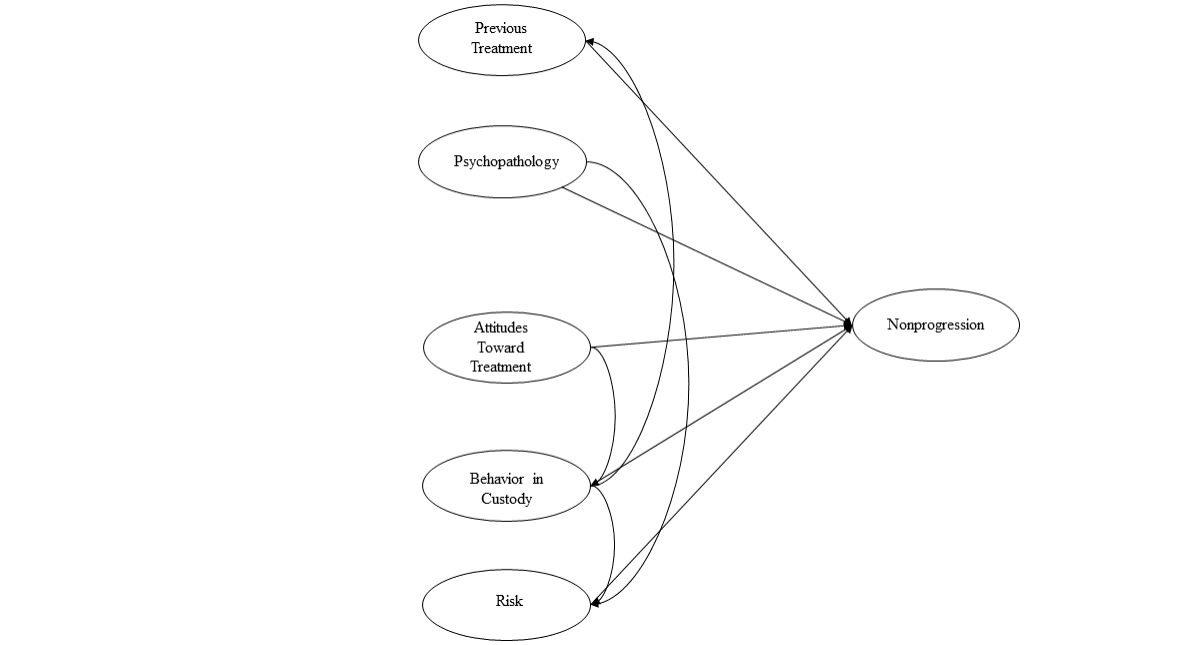
Proposed latent structure of factors predicting failure to progress.

**Figure 2:**
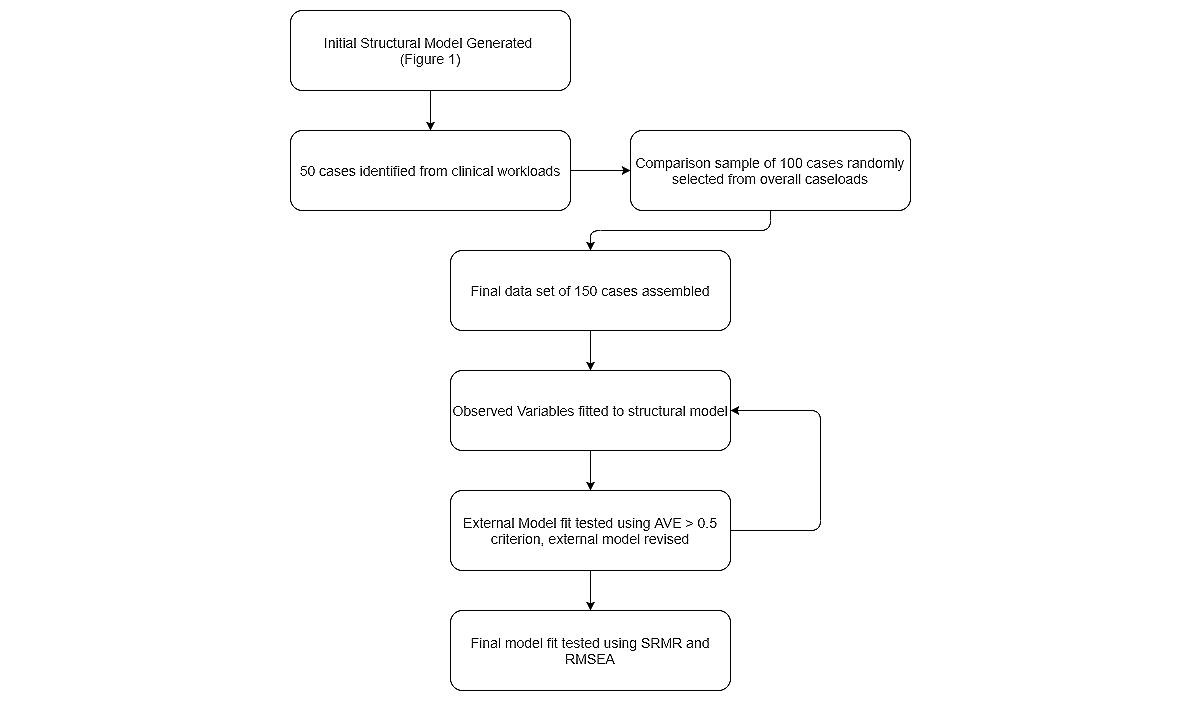
Visualization of data analysis plan. AVE: average variance extracted; RMSEA: root mean square error of approximation; SRMR: standardized root mean square residual.

The model was built using the plspm package [44] in the R statistical programming environment (R Foundation for Statistical Computing) [45]. The observed exogenous variables (see Multimedia Appendix 1 for the outer model) previously described were assessed for their loading onto six latent variables (psychopathology, risk, previous treatment, previous behavior in prison, attitudes toward treatment, and nonprogression). The latent variables create the inner model, and the variables were connected using clinical knowledge and theory (Figure 1). For example, risk models used in OPD services understand attitudes toward treatment are linked to risk, but attitudes toward treatment are also linked to likelihood of engaging in treatment. A conceptual decision was made to treat both *psychopathology* and *attitudes toward treatment* as exogenous concepts although the two are undoubtedly related [46]. However, the SEM approach enforces directionality of relationships (eg, personality must either inform attitudes or vice versa), which was not deemed appropriate here, so the link was omitted. Figure 2 outlines the analysis plan visually.

Based on the coefficients observed from the PLS-SEM model, we considered possible approaches to addressing risk factors identified as most important in predicting failure to progress in the Discussion section.

## Results

### Characteristics of Nonprogression Group Versus Control Group

An objective of the study was to describe the nonprogression sample’s demographic, offending, and clinical characteristics alongside referral information to identify common features of the group. To do this, the nonprogression group was compared to the control group using chi-square tests of difference (or *t* tests where appropriate) to understand the key differences across the samples. Unadjusted *P* values are reported throughout; however, following Bonferroni correction, a “true” significance threshold of *P*<.05 / Y = 46 = *P*<.001 could be considered. Table 1 presents some key demographic information about age, ethnicity, relationship status, and index offences across the sample. Supplementary descriptive summary Tables were also created (Tables S1-S5 in Multimedia Appendix 1).

**Table 1:** Descriptive statistics according to nonprogression and control group.

Demographics		Nonprogression (n=50)	Control cases (n=100)	*t* test (*df*)		Chi-square (*df*)		*P* value	
Age (years), mean (SD)		41.66 (11.12)	40.73 (11.20)	–0.48 (148*)*		N/A^a^		.63	
**Ethnicity, n (%)**					N/A		3.42 (4)		.49
	White	25 (50)	55 (55)						
	Non-White	25 (50)	45 (45)						
**Relationship status, n (%)**					N/A		14.34 (4)		.006
	Single	43 (86)	71 (71)						
	Other	7 (14)	29 (29)						
**Index offence, n (%)**					N/A		17.88 (7)		.01
	Violent offence	32 (64)	70 (70)						
	Sexual offence^b^	13 (26)	8 (8)						
	Other	5 (10)	22 (22)						

^a^N/A: not applicable.

^b^This combines adult and child sexual offences.

#### Demographics and Offending

Overall, the nonprogression group was similar to the control group in ethnicity. The groups differed in marital status, with a slightly higher proportion of singleness (43/50, 86% vs 71/100, 71%) in the control group (*χ*^2^_4,150_=14.34; *P*=.006). The nonprogression group had committed more adult sexual offences (11/50, 22% vs 5/100, 5%), was given more indeterminate sentences for public protection (IPP; *χ*^2^_5,150_=18.90; *P*=.001), and was detained in category B security prisons more frequently than the control group (*χ*^2^_6,150_=40.59; *P*<.001; Table S1 in Multimedia Appendix 1).

#### Attitude Toward Treatment and Behavior in Custody

There was a significant difference in the number of adjudications between the control group (mean 7.52, SD 14.43) and the nonprogression group (mean 15.88, SD 22.58; *U*=1537.5; *P*=.003). The groups also significantly differed in the frequency of time spent in long-term segregation (*χ*^2^_1,150_=10.34; *P*=.002), with the nonprogression group being in long-term segregation more. The nonprogression group was also more likely to misbehave during their sentence (eg, having contraband; *χ*^2^_1,150_=8.68; *P*=.004) and commit a further offence while in custody than the control group (*χ*^2^_6,150_=15.73; *P*=.006). The control group was significantly more motivated toward treatment than the nonprogression group (*χ*^2^_1,150_=30.92; *P*<.001), whereas the nonprogression group displayed more attitudes of having no hope (*χ*^2^_1,150_=8.29; *P*=.007; Table S2 in Multimedia Appendix 1).

#### Psychopathology and Risk

The nonprogression group had significantly higher levels of diagnosis or traits of antisocial (*χ*^2^_1,150_=7.09; *P*=.009) and borderline (*χ*^2^_1,150_=3.73; *P*=.04) personality disorders as well as psychopathy (*χ*^2^_1,150_=16.05; *P*<.001) and learning difficulties (*χ*^2^_1,150_=8.29; *P*=.007). The nonprogression group was more likely to be rated as “high risk” on the OGRS (*χ*^2^_3,150_=8.46; *P*=.03) and scored higher on the HCR-20 (control mean 24.44, SD 5.08; nonprogression mean 28.29, SD 4.68; t_65_=–3.161; *P*=.002), suggesting the nonprogression group displays greater risk (Tables S3-S4 in Multimedia Appendix 1).

#### Previous Treatment and Refusal to OPD Services

The nonprogression group had historically engaged with little treatment aside from offending behaviour programs. Nearly half the nonprogression group had been referred to an OPD medium secure unit or a PIPE and been refused. Around one-third of the sample had been referred to a prison TC and been refused.

### Predicting Refusal by OPD Services (PLS-SEM)

**Figure 3:**
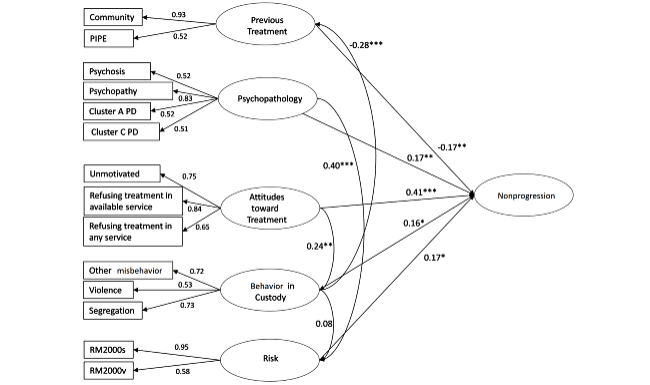
Path diagram showing coefficients of the inner and outer structural model. PD: personality disorder; PIPE: psychologically informed planned environment. *P<.05; **P<.01; ***P<.001.

The next objective of the study was to develop a causal model of service refusal using a SEM method. To do this, we followed a formative approach, proceeding from a theoretical model of likely causal drivers for nonprogression (Figure 1) that we then operationalized with observed variables taken from the available OPD data (Figure 3). For example, the *psychopathology* latent construct was formed from the observable variables of psychopathy; psychotic disorder; and cluster A, B, and C personality disorders.

First, the overall model fit of the estimated model was assessed using the standardized root mean square residual (SRMR). The SRMR value of 0.1 showed an adequate fit of the model [47]. Next, indicator loadings for observed variables on latent constructs were assessed. For indicator loadings to be at an accepted level across the model, some individual indictors were grouped (*cluster A personality disorder* was created by combining paranoid, schizoid, and schizotypal personality disorder, and *cluster C personality disorder* was created from avoidant, dependent, and obsessive-compulsive personality disorder). The loadings in the model were all reviewed, and accepted loading coefficients all fell above 0.5. Observed variables removed due to insufficient indicator loadings included ethnicity, marital status, learning difficulty, denial of offence, no hope toward treatment, abscond or escape, serious further offence in custody, previous treatment in medium secure health, TCs or offender programs, OASys personality disorder screen number, or OGRS and OVP risk scores.

Next, the internal consistency was assessed through the composite reliability of observed variables within each latent construct. All variables fell near or above the recommended threshold of 0.7 (Table 2). Average variance extracted values over 0.5 indicate the construct explains at least 50% of the variance of its items.

**Table 2:** Reflective constructs assessment of composite reliability and convergent validity according to each latent construct.

Reflective constructs	Composite reliability	Average variance extracted
Psychopathology	0.69	0.37
Attitudes	0.88	0.65
Behavior in custody	0.70	0.44
Previous treatment	0.71	0.57
Risk	0.76	0.62

Finally, the discriminant validity was investigated to understand the extent to which the constructs are empirically distinct from other constructs in the model. To assess this, cross-loadings of observed variables on all latent constructs were reviewed. All the cross loadings were higher in value on their relative latent variable, compared to the other latent constructs, meaning there was no issue of discriminant validity. Table S5 in Multimedia Appendix 1 displays the cross-loadings for the discriminant validity check.

The structural model assessment looks at the exogenous and endogenous latent variables through evaluation of the *R*^2^. The *R*^2^ value represents the coefficient of determination and shows the amount of variance of endogenous latent variables explained by the model. The model testing nonacceptance to OPD services (ie, nonprogression) had an *R*^2^ value of 0.45. Cohen et al [48] suggest a good model should have an *R*^2^ value greater than 0.26, which is met and shows the model provides a substantial amount of explained variance in the pathway of nonprogression. The remaining models of risk, previous treatment, and behavior in custody fell below the recommended *R*^2^ threshold (0.18, 0.08, and 0.06, respectively) and therefore should be interpreted with caution.

All specified path coefficients between latent variables were inferred from the *β* value. *β* shows the strength of an effect from the exogenous to the endogenous latent variables. Higher *β* values show stronger effects. The *β* value is then tested for significance through the *t* test. Effect sizes of individual latent variables were calculated using Cohen *f*^2^ [49], which indicates the relative influence of the variable within the overall SEM model.

Table 3 shows the structural model assessments. The most influential factor in nonprogression was attitude toward treatment (*β*=.41; *P*<.001; *f*^2^=0.25). A negative attitude toward treatment, such as refusing treatment in an available service or being unmotivated, was predictive of not progressing with a moderately strong effect size. Behavior in custody (*β*=.16; *P*=.02; *f*^2^=0.04), previous treatment (*β*=–.17; *P*<.001; *f*^2^=0.05), risk (*β*=.17; *P*=.02; *f*^2^=0.04), and psychopathology (*β*=.17; *P*=.01; *f*^2^=0.04) provided significant prediction of nonprogression, however, with weak effect sizes.

The remaining model assessments show psychopathology significantly predicted the level of risk with a moderate effect size (*β*=.40; *P*<.001; *f*^2^=0.19); however, behavior in custody did not predict level of risk (*β*=.08; *P*=.24; *f*^2^=0.01). Behavior in custody significantly predicted previous treatment with a weak effect (*β*=–0.28; *P*<.001; *f*^2^=0.09; ie, more *poor behavior* being related to less prison treatment). Finally, attitudes toward treatment significantly predicted behavior in custody (*β*=.24; *P*=.01; *f*^2^=0.06) but with a weak effect size. Due to the small *R*^2^ sizes of the risk, previous treatment, and behavior in custody models, only a small proportion of variance was explained.

Figure 3 displays the overall model including measured and latent variables with the coefficients and the significant pathways highlighted. The figure shows how the measured variables load onto the latent variables and the strength of effect the latent variables have in explaining nonprogression.

**Table 3:** Structural model assessment.

Model			Overall model *R*^2^		Path coefficient (*β*)		*P* value		*f^2^^a^*
**Nonprogression**			0.45						
	Behavior in custody			.16		.02		0.04	
	Previous treatment			–.17		.001		0.05	
	Risk			.17		.02		0.04	
	Psychopathology			.17		.01		0.04	
	Attitudes			.41		.001		0.25	
**Risk**			0.18						
	Psychopathology			.40		.001		0.19	
	Behavior in custody			.08		.24		0.01	
**Previous treatment**			0.08						
	Behavior in custody			–.28		.001		0.09	
**Behavior in custody**			0.06						
	Attitudes			.24		.01		0.06	

^a^Suggested interpretation of *f^2^* effect sizes.

## Discussion

### Principal Findings

In this paper, we identified and described a sample of offenders who had not progressed on the OPD pathway to understand the common features of this group. The nonprogression group had more sexual offences, were more likely to be on an IPP sentence and in a category B prison, received more adjudications, spent more time in segregation, had more general misbehavior, were assessed as being high risk, had more traits of antisocial and borderline personality disorder as well as psychopathy, were less likely to have engaged with treatment previously, and displayed more attitudes of hopelessness toward treatment.

The features of this group correspond to the literature that has identified factors related to nonengagement with treatment. For example, several personality disorder diagnoses [17], low persistence [18], less previous treatment [12], and being assessed as high risk [5]. Furthermore, the sample of offenders not progressing on the OPD pathway are also characterized by their sexual offending history, IPP sentence type, and prison security category.

Following this, we built a complex regression model to understand the causal drivers by which individual offenders and ex-offenders identified for entry into the UK OPD pathway fail to progress within the pathway. The model showed that, aside from static factors such as risk not amenable to intervention; negative attitudes to treatment; and psychopathology including psychopathy, psychosis, and co-occurring personality disorders were the main significant drivers of failure to progress. Completing a treatment program previously, either in the community or a PIPE was negatively associated with nonprogression.

The regression analyses indicated that limited motivation for treatment was the principal driver of being refused from OPD services. Lacking motivation for treatment, a factor already identified as a problem within populations with a likely diagnosis of personality disorder [50,51], remains a problem within the OPD pathway. Various programs offered to offender populations have attempted to address this issue of low motivation through a psychoeducational approach [52,53]; however, this approach can be resource-intensive and has not been subject to rigorous evaluation.

First, low treatment motivation also predicted problematic institutional behavior, including more custodial adjudications and having spent protracted periods in segregation, and this behavior in turn also predicted service refusal. It is likely that presenting in this way would increase the likelihood of cases struggling to meet behavioral stability criteria to enter many services. Although the relationship between problematic custodial behavior and service refusal was not strong, the results still emphasize that services aiming to support individuals on the OPD pathway need to be able to receive men with patterns of challenging behavior and contain and manage ongoing episodes, without this resulting in treatment termination.

Second, the outer loadings within the SEM model suggest that the single most influential factor was psychopathy or psychopathic disorder, which has long been acknowledged as a limiting factor for treatment and rehabilitation [54]. It could be argued that psychopathic offenders are not best served on a pathway that caters for offenders with personality disorder in the broader sense of the diagnosis, as their needs are known to be different [55]. Treatment programs for this group may therefore need to use more flexible models, which work hard to sustain engagement and rely less on prosocial motivation. An emphasis on promoting positive lifestyle changes in areas of criminogenic need may also be more successful than using traditional therapeutic approaches, which focus on the treatment of maladaptive personality traits [56].

Finally, our results suggest some reasons for cautious optimism. The final finding that treatment completion was negatively associated with nonprogression suggests that nonprogression is a single “hurdle,” and once overcome, that is to say, once an offender successfully engages with a rehabilitative program, they are less likely to become stuck in the future. A possible conclusion from this is that multiple light-touch efforts to engage offenders in rehabilitation programs from an early stage might be a better approach than a single *high stakes* pathway where failure is an *end point*. Some previous treatment approaches for offenders at high risk of reconviction have adopted this approach, with repetition of short duration intensive treatments viewed as a necessary part of progression for some offenders [57].

### Strengths and Limitations

This paper has two key strengths. First, we adopted an uncommon modeling approach that has the potential to show not just predictive associations but also causal links between variables. Causal models are crucial in understanding and managing risk [58], as they can distinguish between predictive associations that are of academic interest but essentially uninformative to the intervention (eg, age or gender) and causal associations where an intervention could potentially be targeted (eg, attitudes to treatment). The PLS-SEM approach allows the use of latent variable modeling to separate confounding or static variables from the model and focus on associations that have clinical meaning. By constructing this model, we have suggested that, in this case, the most important factor in avoiding progression failure is a dynamic risk factor (attitudes to treatment), which has shown amenability to treatment in previous studies.

Second, the sample size identified was more than acceptable for this kind of analysis; Hair et al [37] suggest at least 10 cases per regression estimate in the *busiest* latent variable, which in this case would suggest 70 cases based on the *behavior in custody* variable, which has four endogenous and three exogenous links. In PLS-SEM, larger sample sizes provide important additional generalizability of the models beyond local contexts, which we are claiming here.

However, there were limitations to this paper. First, although the fit statistics for our analysis, which indicate how well our model reflected the structure of the data, were mostly acceptable, they were not unequivocal in their support of the model. For example, an SRMR of less than 0.08 is generally considered a *good fit* [59], but our model did not reach this threshold, although it was close (SRMR=0.1), and statisticians encourage some flexibility in interpretation of these statistics (eg, Hair et al [60] suggest a threshold of 0.12). Second, although the model was able to account for some heterogeneity in demography, psychopathology, and risk between the nonprogression and comparison groups within our sample, the number of differences between the two groups was large and included complex categorical variables such as index offence. Only a larger study using a matching approach (eg, propensity score matching) would truly be able to account for these differences and establish a generalizable model. Third, there was a large amount of missing data for some variables within the model, specifically risk as measured by the HCR-20, which is a standard risk assessment for forensic clients within health settings but is completed by only a small number of prison settings. As the PLS-SEM approach does not allow missing data, this measure was not included in the final model, and therefore, some latent constructs such as *risk* may not be as well specified as we would have hoped.

### Clinical and Practical Implications

The findings of the study are informative about those at the more extreme end of the spectrum of the OPD strategy’s target group, certainly in terms of severity of personality disturbance and risk to others—both in custody and in the community. These results point to the need for a more particular focus on this group in a number of possible ways. First, efforts should be made to identify and target these individuals early in their sentence, before antiauthoritarian attitudes and hopelessness have become entrenched. Second, it could be beneficial to develop a small number of OPD pathway services specifically focused on developing more sophisticated approaches to addressing low treatment motivation. This is known, clinically, to be multifactorial, involving a lack of self-belief, lack of trust, and high levels of distress [61]. Additionally, the role of high epistemic vigilance, or these individuals’ profound reluctance to consider new knowledge and experiences—gained, crucially, through relating to others—as safe and trustworthy, is likely to be relevant let alone worth integrating into their lives [62]. Such services could usefully rely on creative approaches that seem to reduce interpersonal and internal-based threat in the therapeutic encounter, such as art psychotherapy [63]. Peer mentors, especially those who have themselves succeeded in progressing after long years in segregation or otherwise in a state of protest, would also provide a credible means of accessing and addressing ambivalence. Finally, such services will need the flexibility to “weather” periods of disengagement and defiance without rushing to deselect.

### Future Perspectives

This study provides useful information to ensure the OPD pathway is adequately providing for those who need its services. A recommendation from this study would be for the OPD pathway to consistently use pretreatment motivation interventions to effectively engage more offenders. However, these interventions also need evaluation to understand their effectiveness and utility. The study also highlights the difficulty that psychopathic disorder presents in the OPD pathway. Future research should focus on this subsample within the OPD pathway to understand their specific treatment needs and whether they are best served with the pathway approach.

### Conclusion

In this study, we identified a group of individuals for whom the pathway approach was not working due to their refusal into any OPD service, despite referrals to these services. The characteristics of this group were compared to a broader caseload (who have been accepted into OPD services), and PLS-SEM was used to understand causal drivers for nonprogression on the OPD pathway. Previously unknown characteristics of offenders on the OPD pathway who are not progressing have now been described. Furthermore, causally modeling factors involved in nonprogression has shown the current OPD pathway struggles to account for those who are lacking in motivation toward treatment alongside those with psychosis, psychopathy, and co-occurring personality disorder.

## Appendix

Multimedia Appendix 1 Supplementary materials.

## Abbreviations

CJS: criminal justice system
HCR-20: Historical Clinical Risk
IPP: indeterminate sentences for public protection
LPP: London Pathways Partnership
NHS: National Health Service
NPS: National Probation Service
OASys: Offender Assessment System
OBP: offender behavior program
OGRS: Offender Group Reconviction Scale
OPD: Offender Personality Disorder
OVP: OASys Violence Predictor
PIPE: psychologically informed planned environment
PLS-SEM: partial least squares structural equation modeling
RM2000: Risk Matrix 2000
RNR: risk needs responsivity
SEM: structural equation modeling
SRMR: standardized root mean square residual
TC: therapeutic community
