# Supplementary material for: Offenders With Personality Disorder Who Fail to Progress: A Case-Control Study Using Partial Least Squares Structural Equation Modeling Path Analysis
Source: JMIRx Med. 2021 Oct 29;2(4):e27907. doi: 10.2196/27907 (PMC10414335; doi:10.2196/27907)

**Multimedia Appendix 1.** Supplementary materials.

**Table S1.** Descriptive Statistics of Demographic and Offending Characteristics for Non-progression and Control Group.

| *Demographics and Offending Characteristics* | *Non-progression (N= 50)* | *Control Cases (N = 100)* | *Test Statistic* | *P-value* |
| --- | --- | --- | --- | --- |
| Age (Years) *(M, SD)* | 41.66 (11.12) | 40.73 (11.20) | -.48 | .632 |
| Ethnicity (*%)* |  |  | 3.42 | .490 |
| White | 50 | 55 |  |  |
| Mixed | 8 | (12 |  |  |
| Asian/ Asian British | 4 | 5 |  |  |
| Black/ Black British | 36 | 28 |  |  |
| Chinese/ Other | 2 | - |  |  |
| Marital Status (*%)* |  |  | 14.34 | .006 |
| Married | - | 3 |  |  |
| Separated/ Divorced | 4 | 77 |  |  |
| Single | 86 | 71 |  |  |
| Other | 4 | - |  |  |
| Unknown | 6 | 19 |  |  |
| Index Offence (*%)* |  |  | 17.88 | .013 |
| Murder/ Manslaughter | 14 | 22 |  |  |
| GBH/ Serious Assault | 30 | 34 |  |  |
| Armed Robbery | 16 | 14 |  |  |
| Arson | - | 4 |  |  |
| Sexual Offence (adult) | 22 | 5 |  |  |
| Sexual Offence (child) | 4 | 3 |  |  |
| Kidnapping/ False imprisonment | 4 | - |  |  |
| Other | 10 | 18 |  |  |
| Victim of Index Offence (*%)* |  |  | 10.50 | .311 |
| Spouse/ Partner | 6 | 14 |  |  |
| Parent | - | 3 |  |  |
| Own child/ stepchild | - | 2 |  |  |
| Other relative | - | 4 |  |  |
| Friend/ Acquaintance | 24 | 20 |  |  |
| Adult Stranger | 56 | 41 |  |  |
| Child Stranger | 2 | 1 |  |  |
| Professional | 8 | 6 |  |  |
| Other | 4 | 9 |  |  |
| Previous Offending (*%)* |  |  |  |  |
| Violent Offending | 78 | 79 | 0.20 | .888 |
| Sexual Offending | 28 | 12 | 5.96 | .015 |
| Robbery | 12 | 16 | 0.43 | .514 |
| Acquisitive | 78 | 71 | 1.41 | .495 |
| Drug related offence | 32 | 31 | 0.02 | .901 |
| Arson | 65 | 9 | 0.41 | .523 |
| Stalking | 2 | 1 | 0.25 | .615 |
| Other | 100 | 76 | 2.39 | .302 |
| Current Sentence (*%)* |  |  | 18.90 | .002 |
| Life | 32 | 33 |  |  |
| IPP | 48 | 22 |  |  |
| 11-20 years | 2 | 3 |  |  |
| 6-10 years | 12 | 10 |  |  |
| < 5 years | 4 | 31 |  |  |
| Other | 2 | 1 |  |  |
| Current Security Cat (%) |  |  | 40.56 | <.001 |
| A | 2 | 4 |  |  |
| B | 48 | 21 |  |  |
| C | 42 | 18 |  |  |
| D | 1 | 8 |  |  |
| Hospital Order Medium Secure | 2 | 3 |  |  |
| Community | - | 45 |  |  |
| Deported | - | 1 |  |  |
| Recall (%) |  |  | 5.30 | .258 |
| None | 56 | 56 |  |  |
| Once | 30 | 28 |  |  |
| Twice | 6 | 14 |  |  |
| Three or more time | 2 | 1 |  |  |
| Not known | 36 | 11 |  |  |
|  |  |  |  |  |
|  |  |  |  |  |

| *Measure* | *Non-progression (N= 50)* | *Controls (N = 100)* | *Test Statistic* | *P-value* |
| --- | --- | --- | --- | --- |
| Attitudes towards treatment (%) |  |  |  |  |
| Denial of offence | 918 | 13 | .666 | .415 |
| Denial of disorder | 4 | 2 | .514 | .474 |
| No hope | 16 | 3 | 8.29 | .004 |
| Refusing treatment in available service | 12 | 3 | 4.79 | .029 |
| Only accepting treatment in a specific service | 40 | 5 | 29.40 | <.001 |
| Unable to engage | 10 |  | 3.24 | .072 |
| Refusing to engage | 14 | 4 | 4.91 | .027 |
| Motivated | 14 | 62 | 30.92 | <.001 |
| Not Known | 16 | 9 | 1.63 | .202 |
| Behaviour in Custody |  |  |  |  |
| Adjudications (*M*, *SD)* | 15.88 (22.58) | 7.52 (14.43) | 1537.5^a^ | .003 |
| Previous Escape/ Abscond (*%)* | 12 | 13 | 0.30 | .862 |
| Previous riot/ rooftop protest (*%)* | 2 | 1 | 0.25 | .615 |
| Previous long-term/ repeated segregation (*%)* | 34 | 12 | 10.34 | .002 |
| Previous serious institutional violence (*%)* | 50 | 30 | 5.74 | .027 |
| Previous other institutional misbehaviour (*%)* | 90 | 68 | 8.68 | .004 |
| Charged with a further offence (*%)* | 36 | 22 | 15.73 | .006 |

**Table S2.** Descriptive Statistics of Attitudes Towards Treatment and Behaviour in Custody for Non-progression and Control Group

^a^t-test statistic

**Table S3.** Descriptive Statistics of Psychopathology Characteristics for Non-progression and Control Group.

| *Psychopathology* | *Non-progression (N = 50)* | *Control Cases (N= 100)* | *Test Statistic* | *P-value* |
| --- | --- | --- | --- | --- |
| Personality Disorder/ Traits (*%)* |  |  |  |  |
| Anti-social | 62 | 39 | 7.09 | .008 |
| Borderline | 32 | 18 | 3.73 | .044 |
| Paranoid | 20 | 9 | - | - |
| Narcissistic | 12 | 10 | - | - |
| Avoidant | 2 | 4 | - | - |
| Dependent | 4 |  | - | - |
| Schizoid | 4 | 1 | - | - |
| Schizotypal | 2 | - | - | - |
| Histrionic | - | - | - | - |
| Obsessive-Compulsive | 4 | - | - | - |
| Not Specified | 26 | 33 | - | - |
| Psychopathy | 26 | 4 | 16.05 | .001 |
| Other Psychopathology (*%)* |  |  |  |  |
| Psychosis | 12 | 6 | 1.63 | .202 |
| Learning Difficulty | 16 | 3 | 8.29 | .007 |
| Autistic Spectrum Disorder | 4 | 1 | - | - |
| Organic Brain Injury | - | 1 | - | - |
| Depression | 24 | 28 | 0.27 | .602 |
| Substance Misuse | 78 | 67 | 1.95 | .163 |
| PTSD | 6 | 7 | - | - |
| Severe and deliberate self-harm | 36 | 23 | 2.84 | .092 |

**Table S4.** Descriptive Statistics of Risk Measures for Non-progression and Control Group.

| *Risk Measures* | *Non-progression (N = 50)* | *Control Cases (N = 100)* | *P-value* |
| --- | --- | --- | --- |
| OASys Risk Measures |  |  |  |
| OASys ASPD Trigger (+7) (*%*) | 74 | 771 | .796 |
| OASys ASPD Score (*M, SD*) | 7.69 (1.31) | 7.07 (1.83) | .285 |
| OGRS Category (*N* = 149)^a^ |  |  |  |
| Average Score (*M, SD*) | 57. 43 (23.09) | 54.80 (19.39) | .467 |
| Low risk (*%*) | 34 | 40.4 |  |
| Medium Risk (*%*) | 30 | 43.4 |  |
| High Risk (*%*) | 30 | 17.2 | .030 |
| Very High Risk (*%*) | 4 | - |  |
| OVP Category (*N* = 149)^a^ |  |  |  |
| Average Score (*M, SD*) | 42.08 (20.67) | 36.70 (16.51) | .088 |
| Low risk (*%*) | 34 | 38.4 |  |
| Medium Risk (*%*) | 42 | 53.5 |  |
| High Risk (*%*) | 18 | 9 |  |
| Very High Risk (*%*) | 4 | - |  |
| RM2000s (*N* = 30)^b^ |  |  |  |
| Low risk (*%*) | 5 | 30 |  |
| Medium Risk (*, %*) | 35 | 30 |  |
| High Risk (*%*) | 50 | 40 |  |
| Very High Risk (*%*) | 10 | - |  |
| RM2000v (*N* = 65)^c^ |  |  |  |
| Low risk (*%*) | 3.7 | 7.9 |  |
| Medium Risk (*, %*) | 37.1 | 21.1 |  |
| High Risk (*%*) | 48.1 | 57.9 |  |
| Very High Risk (*, %*) | 11.1 | 13.2 |  |
| HCR-20 (*M, SD)* (*N* = 67) | 28.29 (4.68) | 24.44 (5.08) | .002 |

^a^1 missing control case
^b^N = 20 Non-Progression, N = 10 Control Group
^c^N = 27 Non-Progression, N = 38 Control Group

**Table S5.** Cross Loadings of Variable Loadings to Check Discriminant Validity.

|  | *Latent Variables* | | | | | |
| --- | --- | --- | --- | --- | --- | --- |
| *Observed Variables* | *Pathology* | *Attitude* | *Behaviour in Custody* | *Previous Treatment* | *Risk* | *Stuck* |
| Pathology |  |  |  |  |  |  |
| Cluster A PD | **0.517** | 0.045 | 0.157 | -0.059 | 0.161 | 0.170 |
| PCL-R | **0.829** | 0.139 | 0.148 | -0.162 | 0.328 | 0.327 |
| Cluster C PD | **0.513** | -0.052 | -0.097 | 0.053 | 0.296 | 0.084 |
| Psychosis | **0.519** | -0.014 | 0.067 | -0.108 | 0.171 | 0.104 |
| Attitude |  |  |  |  |  |  |
| Motivated | 0.118 | **0.739** | 0.166 | -0.177 | 0.195 | 0.454 |
| Refusing any available service | 0.019 | **0.848** | 0.252 | -0.147 | 0.107 | 0.443 |
| Refusing treating in available service | 0.028 | **0.645** | 0.058 | -0.083 | -0.004 | 0.179 |
| Behaviour in Custody |  |  |  |  |  |  |
| Previous repeated segregation | 0.095 | 0.220 | **0.716** | -0.205 | 0.073 | 0.263 |
| Previous violence | 0.095 | 0.083 | **0.4944** | -0.036 | -0.016 | 0.196 |
| Other | 0.074 | 0.151 | **0.745** | -0.258 | 0.158 | 0.241 |
| Previous Treatment |  |  |  |  |  |  |
| Community treatment | -0.113 | -0.169 | -0.298 | **0.987** | -0.194 | -0.342 |
| PIPE | -0.084 | -0.127 | -0.081 | **0.504** | -0.197 | -0.179 |
| Risk |  |  |  |  |  |  |
| RM2000s | 0.412 | 0.155 | 0.135 | -0.244 | **0.948** | 0.382 |
| RM2000v | 0.172 | 0.092 | 0.067 | -0.099 | **0.587** | 0.128 |

Supplementary Figure 1. Outer Model


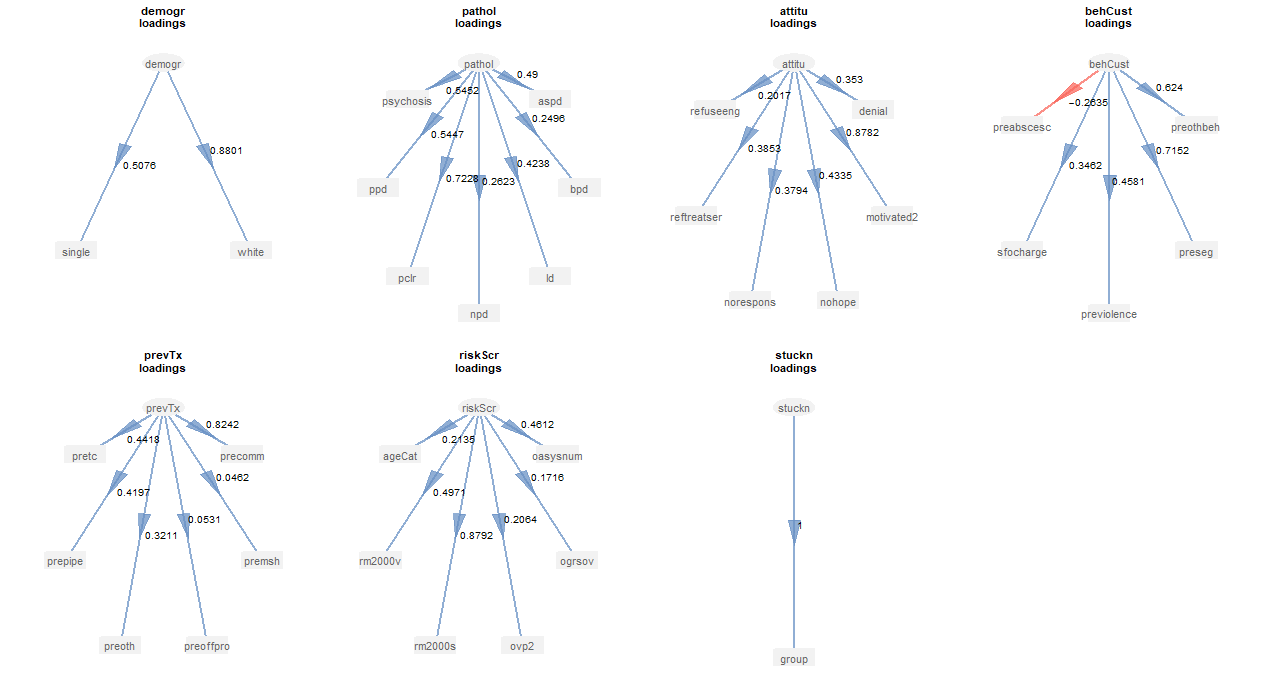

Supplement: Multimedia Appendix 1 [file xmed_v2i4e27907_app1.docx]
